# Tumor suppressor SMAR1 regulates *PKM* alternative splicing by HDAC6-mediated deacetylation of PTBP1

**DOI:** 10.1186/s40170-021-00252-x

**Published:** 2021-04-16

**Authors:** Arpankumar Choksi, Apoorva Parulekar, Richa Pant, Vibhuti Kumar Shah, Ramakrishna Nimma, Priyanka Firmal, Smriti Singh, Gopal C. Kundu, Sanjeev Shukla, Samit Chattopadhyay

**Affiliations:** 1 National Centre for Cell Science, Pune, 411007 India; 2 Indian Institute of Science Education and Research, Bhopal, 462066 India; 3 Kalinga Institute of Industrial Technology (KIIT), Bhubaneswar, 751024 India; 4 Birla Institute of Technology and Science, Pilani – K K Birla Goa Campus, Goa, 403726 India

**Keywords:** SMAR1, PKM1, PKM2, HDAC6, PTBP1, Alternative splicing, Warburg effect

## Abstract

**Background:**

Highly proliferating cancer cells exhibit the Warburg effect by regulation of *PKM* alternative splicing and promoting the expression of PKM2. Majority of the alternative splicing events are known to occur in the nuclear matrix where various MARBPs actively participate in the alternative splicing events. SMAR1, being a MARBP and an important tumor suppressor, is known to regulate the splicing of various cancer-associated genes. This study focuses on the regulation of *PKM* alternative splicing and inhibition of the Warburg effect by SMAR1.

**Methods:**

Immunohistochemistry was performed in breast cancer patient samples to establish the correlation between SMAR1 and PKM isoform expression. Further, expression of PKM isoforms upon modulation in SMAR1 expression in breast cancer cell lines was quantified by qRT-PCR and western blot. The acetylation status of PTBP1 was estimated by immunoprecipitation along with its enrichment on *PKM* pre-mRNA by CLIP in SMAR1 knockdown conditions. The role of SMAR1 in tumor metabolism and tumorigenesis was explored by in vitro enzymatic assays and functional assays upon SMAR1 knockdown. Besides, in vivo tumor formation by injecting adeno-SMAR1-transduced MDA-MB-231 cells in NOD/SCID mice was performed.

**Results:**

The expression profile of SMAR1 and PKM isoforms in breast cancer patients revealed that SMAR1 has an inverse correlation with PKM2 and a positive correlation with PKM1. Further quantitative PKM isoform expression upon modulation in SMAR1 expression also reflects that SMAR1 promotes the expression of PKM1 over tumorigenic isoform PKM2. SMAR1 deacetylates PTBP1 via recruitment of HDAC6 resulting in reduced enrichment of PTBP1 on *PKM* pre-mRNA. SMAR1 inhibits the Warburg effect, tumorigenic potential of cancer cells, and in vivo tumor generation in a PKM2-dependent manner.

**Conclusions:**

SMAR1 regulates *PKM* alternative splicing by causing HDAC6-dependent deacetylation of PTBP1, resulting in reduced enrichment of PTBP1 on *PKM* pre-mRNA. Additionally, SMAR1 suppresses glucose utilization and lactate production via repression of PKM2 expression. This suggests that tumor suppressor SMAR1 inhibits tumor cell metabolism and tumorigenic properties of cancer cells via regulation of *PKM* alternative splicing.

**Supplementary Information:**

The online version contains supplementary material available at 10.1186/s40170-021-00252-x.

## Background

Reprogramming cellular metabolism is one of the key hallmarks of cancer cells. In the presence of oxygen, somatic cells convert glucose to pyruvate by glycolysis and then redirect this pyruvate to the TCA cycle in mitochondria for ATP production. In the absence of oxygen, these cells convert pyruvate to lactate which is an energetically less efficient but relatively fast reaction, whereas highly proliferating cancer cells utilize more glucose and produce more lactate compared with normal cells, independent of the presence or absence of oxygen. This phenomenon is known as the Warburg effect or aerobic glycolysis [1, 2]. Cancer cells reprogram their cellular metabolism by regulating alternative splicing of pyruvate kinase muscle (*PKM*) isoforms [3]. Pyruvate kinase is one of the rate-determining enzymes of glycolysis and facilitates the conversion of phosphoenolpyruvate to pyruvate. There are in total four isoforms of pyruvate kinase enzyme: PKL (pyruvate kinase liver), PKR (pyruvate kinase red blood cells), PKM1, and PKM2. PKL is expressed in the liver, PKR is expressed in the erythrocytes, PKM1 is expressed predominantly in terminally differentiated tissues and PKM2 is highly expressed in the proliferating cells such as cancer cells and stem cells [3, 4]. *PKM* gene contains 12 exons, wherein the incorporation of exon 9 and exon 10 are regulated by mutually exclusive alternative splicing resulting in the expression of PKM1 and PKM2 isoforms, respectively [5, 6]. Exon 9 and exon 10 both code for 56 amino acids attributing to the distinctive regulatory and enzymatic activities of PKM isoforms. PKM1 forms a tetramer and is constitutively active, whereas PKM2 forms a tetramer (enzymatically efficient), as well as a dimer (enzymatically less efficient). Enzymatic activity of PKM2 dimer is influenced by allosteric regulation due to fructose-1,6-bisphosphate levels and interaction with phosphotyrosine-binding proteins [3, 4, 7].

Higher expression of PKM2 provides cancer cells with a metabolic advantage over normal cells. Due to the lower enzymatic activity of PKM2 as compared with that of PKM1, the major amount of glucose present in the cell remains as glycolytic intermediates which provide building blocks, such as amino acids, nucleotides, and fatty acids, to the highly proliferating cancer cells [8]. The remaining glucose gets converted to pyruvate and this pyruvate instead of entering the TCA cycle gets converted to lactate. To meet the high energy and carbon demands these cancer cells utilize more and more glucose compared with normal cells [2]. Apart from its role in glucose metabolism, PKM2 also regulates other cellular processes by getting translocated to the nucleus from the cytoplasm and affecting a variety of signaling pathways leading to oncogenesis [9, 10].

Cancer cells achieve a metabolic edge over normal cells by regulating *PKM* alternative splicing and promoting the expression of PKM2 [3, 8]. hnRNPs (heterogeneous ribonucleoproteins), hnRNP A1, hnRNP A2, and PTBP1 (hnRNP I), are the key regulators of *PKM* alternative splicing [11, 12]. c-Myc binds to promoters of these three genes and regulates their expression [12]. In majority of cancers, high expression of c-Myc promotes the higher expression of these three hnRNPs. When present in abundance, these three proteins bind on the intronic region flanking exon 9 and inhibit the incorporation of exon 9, and as an effect, there is the inclusion of exon 10 of *PKM* gene [13]. Molecular targeting of regulation of *PKM* alternative splicing by these three proteins might prove to be an effective strategy in eradicating the cancer cells [14].

The nuclear matrix is the site for many important nuclear events such as DNA replication, DNA repair, transcription, and post-transcriptional modifications [15, 16]. Post-transcriptional modifications include RNA splicing, RNA capping, and poly-A tail addition. The nuclear matrix is a scaffold around which chromatin folds and many nuclear matrix–binding proteins are involved in chromatin organization [15]. Apart from chromatin compaction, nuclear matrix–binding proteins which are also known as the matrix-associated region (MAR)-binding proteins (MARBPs), actively participate in the regulation of DNA replication, DNA repair, transcription, and post-transcriptional modifications [15, 16]. One such MARBP is SMAR1 (scaffold/matrix attachment region–binding protein 1) [17]. The human homolog of SMAR1 is also known as BANP (BTG3-associated nuclear protein). SMAR1 has been reported to play an important role as a tumor suppressor protein and in the majority of higher grades of cancer, SMAR1 has been reported to be dysregulated [18–24]. *SMAR1* is located on human chromosome 16q24 and loss of heterozygosity (LOH) has been reported for this chromosomal region in various cancers [25–27].

SMAR1 has been reported to colocalize with SC35 which is a marker of nuclear splicing speckles and an important regulator of alternative splicing [28]. Moreover, SMAR1 has an RS domain that facilitates the RNA binding and it interacts with snRNAs which are the core components of splicing machinery [28]. SMAR1 has been reported to regulate splicing of *CD44* variants by deacetylation of Sam68 with help of HDAC6 in breast cancer cell lines. In addition to *CD44* variants, SMAR1 also regulates alternative splicing of *FAS* ligand [28]. ChIP-sequencing study of SMAR1 in HCT116 cells suggests that there are several global gene targets of SMAR1 which are involved in the regulation of RNA processing and alternative splicing [29]. This suggests that SMAR1 might be involved in alternative splicing regulation of other cancer-associated genes. This study focuses on the role of SMAR1 in the regulation of *PKM* alternative splicing via HDAC6-dependent deacetylation of PTBP1 and its implication in inhibition of the Warburg effect and tumorigenesis. Our study thus demonstrates the inhibition of cancer cell metabolism and breast cancer progression by SMAR1 via suppression of oncogenic isoform PKM2.

## Materials and methods

### Cell culture

MCF7, MDA-MB-231, MDA-MB-468, and T47D cells were obtained from the NCCS cell repository, Pune, India. MCF7, MDA-MB-231, and MDA-MB-468 were cultured in DMEM (Gibco) and T47D was cultured in RPMI (Gibco). All the cell lines were supplemented with 10% fetal bovine serum (FBS) (Gibco) and 100 units/ml penicillin and streptomycin (Gibco) and incubated in a humidified 5% CO_2_ incubator at 37 °C.

### Plasmids, siRNAs, and shRNA constructs

3xFlag26-SMAR1 was used to over-express SMAR1 and 3xFlag26-Vector was used as a control. *Silencer*™ Select Negative Control No. 1 siRNA (4390843) and si-RNA against human-SMAR1 (BANP) (s29889) were obtained from Thermo Fisher Scientific Silencer® select siRNA range. shSMAR1-eGFP (ULTRA-3344235) was obtained from TransOMIC and used for shRNA-mediated SMAR1 knockdown. shNon translated-1-eGFP (shNT1) (TLNSU1420) was also obtained from TransOMIC and used as a control for all shRNA-mediated knockdown studies. shHDAC6 clone TRCN0000004839 was used for HDAC6 knockdown [28]. shPKM2 was cloned in pLKO.1-TRC vector and the sequence targeting only PKM2 were obtained from Cortés-Cros et al. [30]. The sequence of shPKM2 used: 5′CCGGCTACCACTTGCAATTATTTGACTCGAGTCAAATAATTGCAAGTGGTAGTTTTTG3′.

### Transfections and treatments

Transfections of various plasmids were done by the use of Polyethylenimine (PEI MAX 40000) (Polysciences, Inc.). In MCF7, transfection was performed at 70–80% confluency and the DNA:PEI ratio used for transfection was 1:3. In MDA-MB-231, reverse transfection was performed in which DNA: PEI mix was added before cell seeding. Tubacin (Sigma), a selective HDAC6 inhibitor, was used at 5 μM concentration for 5 h for optimum HDAC6 inhibition and an equal amount of DMSO was used as vehicle control.

### Cloning of dual reporter *PKM* minigene system

To develop a dual chromatic *PKM* minigene system, eGFP was cloned into mCherry-N1 vector between SalI (NEB) and AgeI (NEB) restriction sites in such a way that both eGFP and mCherry remain in two different frames due to difference in one base pair and eGFP contains stop-codon at the end when in the frame. Further, exon 8–exon 11 of the *PKM* gene along with introns was amplified from *PKM* minigene construct [31] (a kind gift from Dr. Adrian R. Krainer) using Platinum™ SuperFi™ DNA Polymerase (Invitrogen) and cloned into this vector between XhoI (NEB) and NdeI (NEB) sites by Infusion cloning (NEB). One base pair insertion was further introduced in the cloned plasmid in exon 10 by Infusion cloning. Due to one base pair insertion, the incorporation of exon 9 resulted in the expression of mCherry and the incorporation of exon 10 led to the expression of eGFP. The expressions of mCherry and eGFP were analyzed by confocal microscopy.

### Dual reporter *PKM* minigene assay

MCF7 cells were seeded onto a glass coverslip and after 24 h *PKM* minigene along with control or Flag-SMAR1 in a 1:1 ratio was introduced in these cells by PEI-mediated transfection. After 48 h of transfection, cells were fixed with 4% paraformaldehyde for 10 min at room temperature. Subsequently, cells were washed with 1X PBS thrice and the coverslips were mounted in fluoroshield media (Sigma) with DAPI. Cells were observed at × 60 magnification using a Nikon A1plus confocal microscope and images were acquired using Nikon’s NIS-elements imaging software. For eGFP and mCherry fluorescent intensity quantification, the ImageJ software was used. Five independent regions of interest (ROIs) were selected per image covering cells expressing eGFP and mCherry and fluorescence intensity density was measured. Average relative fluorescence densities were calculated for all five ROIs per field and the fold change in eGFP/mCherry ratio was calculated. For statistical significance, three random fields per sample were selected and three independent experiments were performed. Mean ± SD fold change in eGFP/mCherry ratio was calculated for each sample and compared with the control sample.

### Immunohistochemistry

Paraffinized human breast cancer patient samples along with surrounding normal breast tissue were obtained from Ruby Hall Clinic, Pune, India. SMAR1, PKM1, and PKM2 expression were detected using standard immunohistochemical staining procedure. Briefly, after deparaffinization, endogenous peroxidase blockage, and rehydration with decreasing concentrations of ethanol (100%, 95%, and 70%), antigen retrieval was performed by heating the slides in 10 mM sodium citrate buffer (pH 6–7). Further samples were incubated with the antibody of SMAR1 (Bethyl) at 1: 100 dilution, PKM1 (CST) at 1: 100 dilution, and PKM2 (CST) at 1: 300 dilution overnight at 4 °C. After incubation with HRP-conjugated secondary antibodies for 1 h at room temperature, sections were then treated in DAB (3,3′-diaminobenzidine) for 15 min to allow the development of brown precipitate corresponding to the sites of HRP-bound antibodies. Samples were washed and counterstained with hematoxylin for nuclei staining. Tissue sections were observed at × 20 magnification using a Nikon microscope (Eclipse E600) and images were acquired using Nikon’s NIS-elements imaging software.

### Quantitative RT PCR

Total RNA was extracted from cultured MCF7 cells and MDA-MB-231 cells by TRizol^TM^ (Invitrogen) according to the manufacturer’s instruction. RNA was reverse transcribed by MMLV-RT (Invitrogen) as per the manufacturer’s instructions. Amplification reactions were prepared in triplicate using iQTaq SYBR green (Biorad) and amplification was performed on an Eppendorf realplex 2.0 according to the manufacturer’s instruction. The average cycle thresholds from three independent biological replicate samples were calculated as described in Singh et al. with slight modifications [32]. Briefly, the average cycle thresholds from three independent biological replicate samples were normalized to housekeeping control gene *18S rRNA*. Normalization was performed using *18S rRNA* as a normalization control using the formula: [2^ ^(Ct control − Ct target)^]. Along with control gene normalization, constitutive exon (exon 11) normalization was performed for *PKM1* (exons 8–9/9) and *PKM2* (exons 10–11/11) expression analysis. Student’s *t*-test was used to compare expression between two different groups. A list of primers used is given in Table S1.

### Western blotting

Cells were incubated in TNN buffer [50 mM Tris-Cl pH 7.5, 5 mM EDTA, 0.5% NP40, 50 mM NaF, 1 mM DTT, 0.2 mM sodium orthovanadate, 0.5 mM PMSF, 150 mM NaCl and 1× Protease inhibitor cocktail (Thermo Scientific)] for cell lysis and lysates containing an equal concentration of proteins were resolved using SDS-PAGE and transferred onto PVDF membrane. The membranes were incubated with primary antibodies such as anti-SMAR1 (Bethyl-A300-279A), anti-PKM1 (CST-7067), anti-PKM2 (CST-4053), anti-hnRNP A1(CST-8443), anti-hnRNP A2 (Abcam-ab6102), Anti-PTBP1 (Thermo Scientific-32-4800), anti-HDAC6 (CST-7558), anti-HDAC1 (CST-5356), anti-GFP (Proteintech-66002-1-Ig), anti-mCherry (Proteintech-26765-1-AP), anti-β-actin (Sigma-A2228), and anti-Flag tag (CST-14793). This was followed by three washes and incubation with appropriate HRP-conjugated secondary antibodies. Visualization was achieved with ECL substrate (Pierce) and exposure to X-ray films or imaging in Syngene G:BOX Chemi XRQ.

### Antibody cross-linking, co-immunoprecipitation, and sequential co-immunoprecipitation

The majority of Immunoprecipitation (IP) experiments were done via covalently cross-linking the antibody with Protein G Dynabeads (Pierce) to avoid non-specific binding and contamination of immunoglobulin in the immunoprecipitated protein eluates. Approximately 1 μg of antibody was cross-linked with 10 μl of beads. Beads were washed thrice with ice-cold 1× PBS and further incubated with the desired antibody in IP buffer (1× PBS with 0.1% NP-40) containing protease inhibitor cocktail (Pierce). The antibody-bead mixture was incubated overnight at 4 °C. The unbound antibody was removed by three washes of the antibody-bead complex with IP buffer. Further, the antibody-bead complex was incubated with 500 μL of 10 mg/ml of dimethyl pimelimidate (DMP) (Sigma) for 60 min at room temperature with rotation. Fifty microliters of 1 M Tris-Cl pH 8 was added to quench the reaction and incubated for 30 min at room temperature with rotation. Unbound antibody was removed by washing it with 0.2 M glycine pH 3 followed by three washes with IP buffer. The antibody-bead complex was further equilibrated with the IP buffer. These beads were either used immediately or stored at 4 °C for 2–3 days. Protein-bound to the antibody-bead complex was eluted at 95 °C by using SDS loading dye.

For IP experiments, 500 μg of nuclear extracts were pre-cleared with control normal IgG (Sigma) bound Dynabeads G and subsequently incubated for 12 h at 4 °C with Dynabeads G crosslinked with the desired primary antibody. The protein-associated bead complexes were washed thrice with IP buffer and then further eluted with SDS loading dye. The eluates were probed with indicated antibodies.

For sequential IP experiments, nuclear extracts (1 mg) were immunoprecipitated first with 3 μg of anti-SMAR1 antibody. Before proceeding for the second IP, a minor fraction of the eluates was examined for the presence of HDAC6. Subsequently, the eluate was immunoprecipitated with 2 μg of anti-HDAC6. The final eluates were probed with anti-PTBP1 to evaluate the association.

### Anti-acetyl-lysine acetylation assay

For assessing the acetylation status of protein, 1 mg of nuclear extract was incubated with 2 μg of anti-acetyl-lysine antibody (CST- 9441) crosslinked with Dynabeads G at 4 °C with slight mixing for 12 h. Immunocomplexes were washed thrice with 500 μL of IP buffer and further eluted at 95 °C with SDS loading dye. The eluates were loaded onto the SDS-PAGE and immunoblotted with indicated antibodies.

### UV-crosslinking and RNA immunoprecipitation

UV-crosslinking and RNA immunoprecipitation (CLIP) experiment was done via covalently cross-linking the anti-PTBP1 antibody with Protein G Dynabeads (Thermo Scientific) as described in antibody cross-linking. Normal mouse IgG crosslinked beads were used as a negative control. Antibody cross-linked beads were further blocked for 1 h at 4 °C with 100 nM yeast tRNA to avoid non-specific binding with RNA. Cells were washed with ice-cold 1X PBS, UV-irradiated (150 mJ/cm^2^) and harvested and lysed with lysis buffer (100 mM KCl, 5 mM MgCl_2_, 10 mM HEPES—pH 7.0, 0.5% NP40, 1 mM DTT, 100 units/ml RNase Out and Protease inhibitor cocktail) for 10 min on ice. RNase A (1:1000 dilution) and DNase I (Invitrogen) were added to the lysate and incubated at 37 °C for 3 min. 5% lysate was taken as input and TRizol LS^TM^ (Invitrogen) was added for RNA extraction. An equal amount of protein (10 mg) was taken for control and SMAR1 knockdown sample and anti-PTBP1 and normal IgG-conjugated beads were added for immunoprecipitation and incubated on rotation at 4 °C for 3 h. After two washes with wash buffer (50 mM Tris-HCl—pH 7.4, 150 mM NaCl, 1 mM MgCl_2,_ 0.05% NP40) supplemented with RNase inhibitor, an aliquot (10%) of beads was kept as control of immunoprecipitation while the rest was treated with 30 μg of Proteinase K and incubated for 1 h at 55 °C. RNA was then extracted by TRizol LS^TM^ (Invitrogen) and RNA was reverse transcribed by MMLV-RT (Invitrogen) as per the manufacturer’s instructions. Immunoprecipitated fractions and 5% input were analyzed by quantitative real-time PCR in duplicate using iQTaq SYBR green (Biorad) and amplification was performed on a Eppendorf realplex 2.0 and specific primers for PTBP1-binding site on intron 8 of *PKM* were used (sequences mentioned in Table S1). Primer sequence for *PKM* intron 8 PTBP1–binding site was obtained from Chen et al. [13]. The experiment was performed three times and normalization was performed to input using the formula: [2^ ^(Ct input − Ct immunoprecipitation)^]. Fold enrichment was calculated relative to normal mouse IgG control. Resultant fold enrichment was further normalized with densitometry measurement of a western blot for immunoprecipitated PTBP1 protein samples. Student’s *t*-test was used to identify the significance between two different groups.

### Glucose assay

MCF7 cells were transfected with respective shRNA and control shRNA. shRNA-transfected cells were selected with puromycin and an equal number of cells were plated in a 6-well plate. Moreover, MDA-MB-231 cells were seeded in an equal number in a 6-well plate and transfected with Flag-SMAR1 and vector control. After 24 h, the cells were replenished with 10% FBS containing high glucose DMEM (without sodium pyruvate). After 24 h of media replenishment, media was collected and the amount of glucose was calculated with the use of a Glucose Assay Kit (Abcam, ab65333) as per the manufacturer’s protocol by colorimetric method. The amount of glucose present was normalized with the total amount of protein. The glucose utilization was calculated by subtracting the glucose level of samples from that of cell-free media. The percentage of glucose utilization was calculated compared with the control.

### Lactate assay

MCF7 cells were transfected with respective shRNA and control shRNA. shRNA-transfected cells were selected with puromycin and an equal number of cells were plated in a 6-well plate. Moreover, MDA-MB-231 cells were seeded in an equal number in a 6-well plate and transfected with Flag-SMAR1 and vector control. After 24 h, the cells were replenished with 10% FBS containing high glucose DMEM (without sodium pyruvate). After 24 h of media replenishment, media was collected and the amount of lactate was calculated with the use of a Lactate Assay Kit (Abcam, ab65331) as per the manufacturer’s protocol. The amount of lactate present in each sample was normalized with the total amount of protein. The percentage of lactate production was calculated for each sample compared with the control.

### Glucose (2-NBDG) uptake assay

MCF7 cells were transfected with SMAR1 siRNA and control siRNA by lipofectamine RNAiMax^TM^ (Ambion) according to the manufacturer’s protocol. After 24 h of transfection, media was removed and replenished with 10% FBS containing DMEM (without glucose and sodium pyruvate) and incubated at 37 °C for 1 h. 10 μM fluorescent d-glucose analog 2-[N-(7-nitrobenz-2-oxa-1,3-diazol-4-yl)amino]-2-deoxy-d-glucose (2-NBDG) (Invitrogen) was added to culture media and cells were incubated for 1 h at 37 °C. The 2-NBDG uptake reaction was stopped by removing the incubation medium and the cells were washed with ice-cold 1× PBS. 1 μg/ml propidium iodide (PI) was added to distinguish the viable cell population. For each measurement, data from 10,000 single-cell events were collected using FACS Canto II (BD Bioscience). The percentage of 2-NBDG uptake was calculated from mean fluorescence intensity (MFI) compared with the control.

### Cell viability assay

MCF7 cells were transfected with BORIS shRNA and shNT1 was used as a control in six-well culture plates. After the selection of transfected cells with puromycin, cells (4 × 10^3^) were seeded in 96-well culture plates and were cultured for 24 h, 48 h, and 72 h. Cell growth was determined by measuring the conversion of MTT Tetrazolium salt (Sigma) to MTT formazan. In brief, 50 μl of MTT stock solution (5 mg/ml) was added to each well along with 50 μl of 10% FBS containing DMEM and incubated for 4 h. After the incubation time, formazan crystals formed in the cells were solubilized in Isopropanol. The cell viability was measured by Spectramax M5 (Molecular Devices) at an optical density of 570 nm. The percentage proliferation was calculated and cell viability at 0 h for each sample was considered 100%. Compared with 0 h, the percentage of proliferation was calculated for other time points.

### Colony formation assay

MCF7 cells were transfected with respective shRNAs and shNT1 was used as a control. The cells were selected with puromycin and 1 × 10^3^ cells were seeded in the new 6-well plate. After 10 days, cells were fixed using methanol and acetic acid (3:1) for 5 min. After fixation, cells were washed with 1h PBS thrice. After washing cells were stained with 0.05% crystal violet stain, images were taken and colonies formed were counted manually with help of the ImageJ software. The percentage of colony formation was calculated for each sample compared with the control.

### Transwell migration and invasion assay

Transwell chamber: 24-well, 8.0-μm pore membranes (Corning USA) were used according to the manufacturer’s protocol. MCF7 cells were transfected with respective shRNAs and shNT1 was used as a control. After the selection of transfected cells with puromycin, 1x10^5^ cells per well were seeded in the upper chamber in a serum-free medium, and DMEM with 5% FBS was added to the lower chamber as a chemoattractant at the same time. After incubation of 24 h at 37 °C, the cells remaining at the upper surface of the membrane were removed with cotton swabs, and the cells on the lower surface of the membrane are the migrated cells. After fixation with 4% paraformaldehyde and staining with 0.5% crystal violet solution, the cells were observed at 10X magnification using the Nikon microscope (Eclipse Ti2) and images were acquired using Nikon’s NIS-elements imaging software.

The transwell invasion assay was carried out as described above, except that a transwell chamber with Matrigel (Corning, USA) was used and they were pre-incubated at 37 °C after hydration with 100 μL of serum-free medium for 2 h before the cells were seeded onto the membrane, followed by incubation of 48 h at 37 °C. Five fields were randomly captured and the number of migrating/invading cells were quantified manually with the help of Image J software. The percentage of migration/invasion was calculated for each sample compared with the control.

### Wound healing assay

MCF7 cells were transfected with respective shRNAs and shNT1 was used as a control. The cells were selected with puromycin and 1.5 × 10^5^ cells were seeded in each well of a 12-well plate and allowed to grow at 37 °C to form a monolayer. Cells were synchronized by serum starvation for 12 h and scratch was introduced in the middle of the monolayer by a sterile pipette tip, generating a cell-free area of approximately 1 mm in width and cell debris was removed by washing twice with 1× PBS. Three fields per well were imaged in the area where the wound was introduced at 0 h and 24 h. The area of the wound was measured by ImageJ for 0 h and 24 h and the percentage of wound migration was calculated compared with that of the control. All images were taken at × 10 magnification using the Nikon microscope (Eclipse Ti2) with help of Nikon’s NIS-elements imaging software.

### In vivo tumor generation

All mice used in this experiment were bred at the animal resource facility of NCCS, Pune, India. Standard protocols approved and monitored by the Institutional Animal Ethical Committee were followed for this experiment. The MDA-MB-231 cells were transduced with SMAR1-adenovirus and control-adenovirus. One million cells were injected subcutaneously in 6–8 weeks old NOD/SCID mice. After 1 month of injection, mice were sacrificed and tumors were dissected. The volume and weight of tumors were measured and parts of the tumor were utilized for western blot and immunohistochemistry as described in the above section.

### Statistical analysis

All statistical analysis was performed using Microsoft Excel and graphs were plotted using GraphPad Prism7. Data has been represented as mean ± SD. Student’s *t*-test was used to determine the statistical significance of the difference between the groups. The *p* value of < 0.05 was considered significant. *p < 0.05, **p < 0.01 and ***p < 0.001. Images were analyzed and quantified using the ImageJ software.

## Results

### SMAR1 exhibit a negative correlation with PKM2 and a positive correlation with PKM1 expression in breast cancer cells

**Fig. 1:**
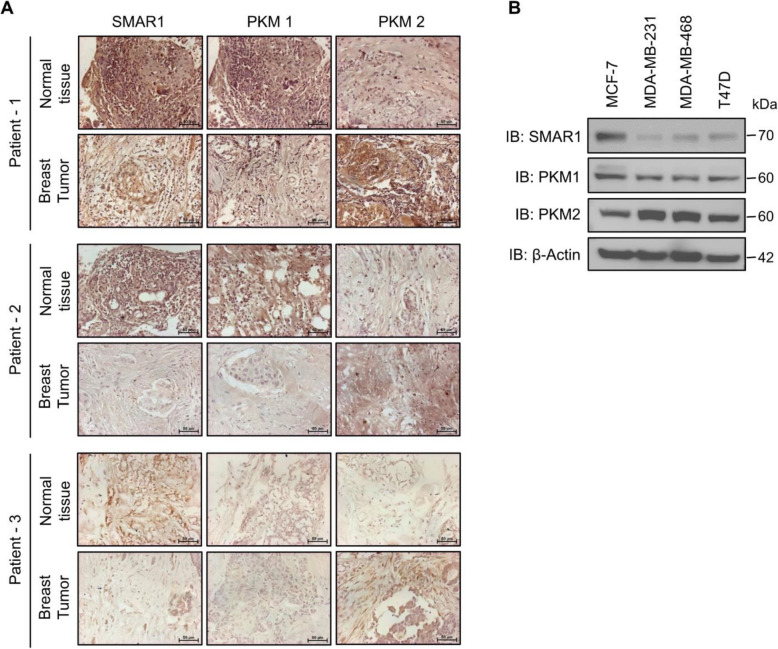
Expression of SMAR1 and PKM isoforms in breast cancer. **a** Expression of SMAR1, PKM1, and PKM2 in paraffinized breast tumor samples compared with normal tissue by immunohistochemistry (*n* = 3). **b** Expression profile of SMAR1, PKM1, and PKM2 in various breast cancer cell lines by western blot

Dysregulation of various tumor suppressor proteins is one of the key features of various cancers [33]. SMAR1 being an important tumor suppressor protein is known to get downregulated in higher grades of breast and colon cancer [21, 23, 24]. LOH of *SMAR1* locus (human chromosome 16q24) has been reported in various cancer [25, 26]. Moreover, Cdc20-mediated proteasomal degradation of SMAR1 has been studied in breast cancer cells [23]. In contrast, upregulation of key oncogenes such as PKM2 gives metabolic as well as tumorigenic advantage to the cancer cells. The majority of normal differentiated cells have a lower expression of PKM2 and in cancer cells, there is an enhanced expression of PKM2 [34]. Expression of SMAR1, PKM2, and PKM1 was measured in breast cancer patient samples and compared with surrounding non-cancerous tissue by immunohistochemistry. IHC staining of SMAR1 revealed that its expression was diminished in tumor samples compared with that of normal control tissue (Fig. 1a). These results correspond with the earlier studies, confirming the downregulation of SMAR1 in breast cancer samples [21, 23]. Moreover, IHC staining of PKM isoforms revealed that PKM2 expression was elevated and PKM1 expression was significantly low in tumor samples compared with that of normal control tissue. These results further comply with earlier reports suggesting a switch in PKM isoform expression between normal tissue and cancer tissue leading to higher expression of oncogenic isoform PKM2 and suppression of PKM1 [3, 32]. Human breast cancer cells such as MDA-MB-231, MDA-MB-468, and T47D harbor LOH for *SMAR1* locus, whereas MCF7 cells lack LOH for this locus [25, 27]. LOH harboring cells have significant downregulation of SMAR1 expression compared with that of MCF7 [28]. Expression profiles of SMAR1, PKM1, and PKM2 in these breast cancer cell lines MCF7, MDA-MB-231, MDA-MB-468, and T47D were found to be correlating with that of the patient samples (Fig. 1b). These results suggest that SMAR1 has an inverse correlation with PKM2 expression whereas it has a direct correlation with PKM1 expression. This indicates that SMAR1 might be promoting the expression of PKM1 and suppressing the expression of oncogenic isoform PKM2 in the non-cancerous tissue.

### SMAR1 regulates *PKM* alternative splicing by promoting the incorporation of exon 9 and suppressing the incorporation of exon 10

**Fig. 2:**
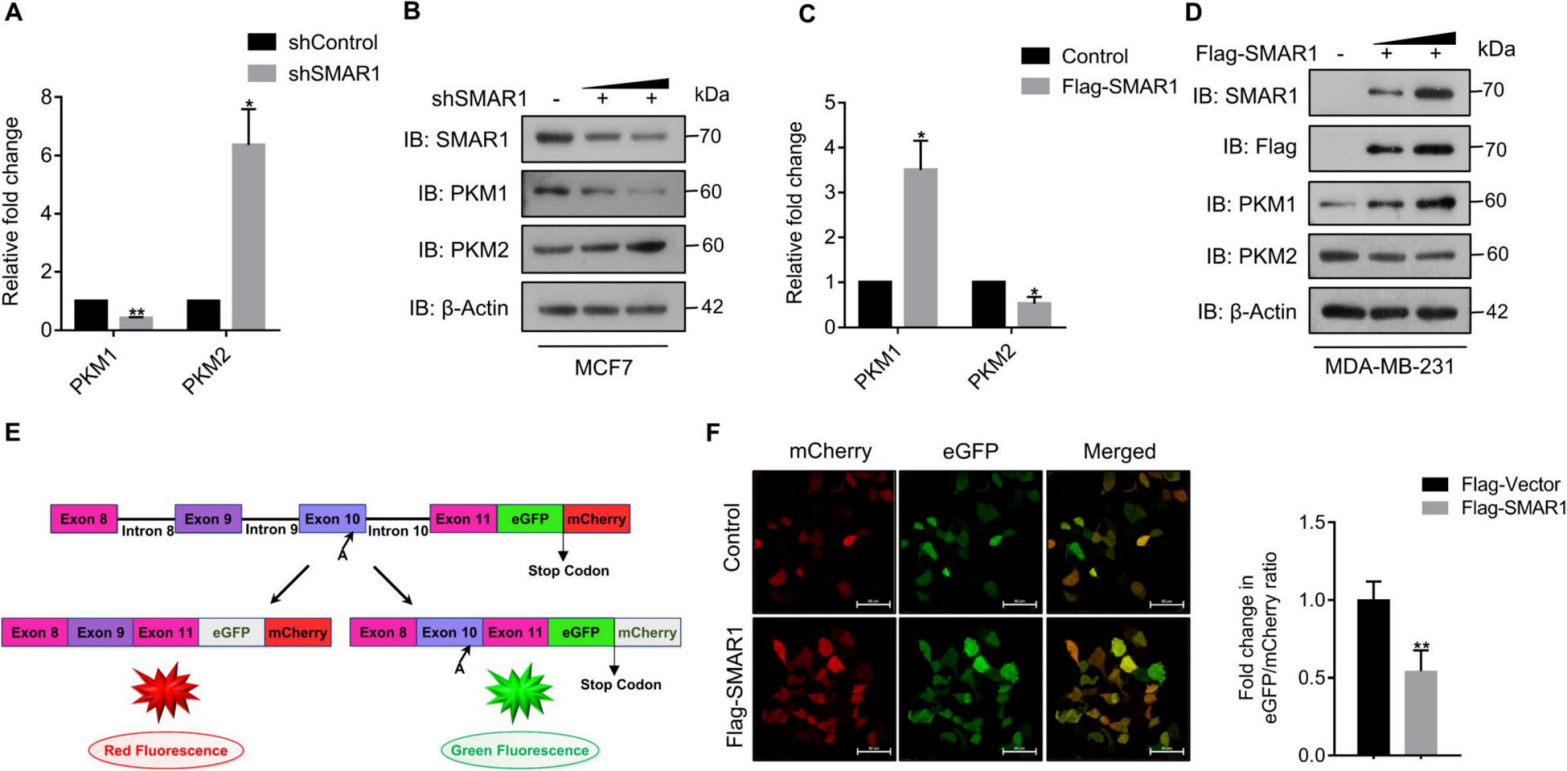
SMAR1 regulates *PKM* alternative splicing. **a** qRT-PCR of PKM isoforms normalized to *18S rRNA* and further normalized to constitutive *PKM* exon (exon 11) upon shRNA-mediated knockdown of SMAR1 in MCF7 (*n* = 3). **b** Expression of PKM isoforms upon shRNA-mediated knockdown of SMAR1 in MCF7 by western blot. **c** qRT-PCR of PKM isoforms normalized to *18S rRNA* and further normalized to constitutive *PKM* exon (exon 11) upon Flag-SMAR1-mediated overexpression of SMAR1 in MDA-MB-231 (*n* = 3). **d** Expression of PKM isoforms upon Flag-SMAR1-mediated overexpression of SMAR1 in MDA-MB-231 by western blot. **e** Schematic representation of dual chromatic *PKM* minigene system. **f** Confocal microscopy to check the expression of eGFP and mCherry in MCF7 cells transfected with *PKM* minigene along with Flag-SMAR1. Relative fluorescence intensity of eGFP and mCherry was quantified with the help of ImageJ and fold change in eGFP/mCherry ratio was calculated for control and Flag-SMAR1 overexpression (*n* = 3). Error bars show mean values ± SD. Differences were considered statistically significant with *p < 0.05, **p < 0.01, and ***p < 0.001; *ns* non-significant difference (*p* > 0.05)

SMAR1 is known to colocalize with one of the key splicing regulators SC35 in nuclear splicing speckles. It also interacts with snRNAs which are the core components of splicing machinery. Moreover, SMAR1 has been reported to regulate alternative splicing of *CD44* variants and *FAS* ligand [28]. On the basis of an inverse correlation between SMAR1 and PKM2 expression as well as a positive correlation between SMAR1 and PKM1 observed in breast cancer, we hypothesized that SMAR1 might play a crucial role in regulating alternative splicing of the *PKM* gene. Based on the expression profile of SMAR1 in various breast cancer cell lines, MCF7 and MDA-MB-231 were used for further experiments. To investigate the role of SMAR1 in the regulation of *PKM* alternative splicing, PKM isoform expression was measured upon shRNA-mediated depletion of SMAR1 expression in MCF7 cells at the RNA level by performing quantitative RT-PCR and at the protein level by western blot. Knockdown of SMAR1 in MCF7 resulted in PKM1 downregulation and increased PKM2 expression at the transcript level (Fig. 2a). Relative fold change in SMAR1 expression upon shRNA-mediated SMAR1 knockdown in MCF7 has been represented in Figure S1A. Further analysis of PKM isoform expression in SMAR1 depleted cells at the protein level suggests diminished expression of PKM1 and upregulation of PKM2 which was further in coherence with RNA expression analysis (Fig. 2b). Moreover, PKM isoform expression was quantified upon ectopic expression of SMAR1 in LOH-containing MDA-MB-231 at the RNA level by qRT-PCR and at the protein level by western blot. Upon Flag-SMAR1 overexpression in MDA-MB-231, PKM1 was observed to be increased and PKM2 was downregulated at the transcript level (Fig. 2c). Flag-SMAR1-mediated overexpression in MDA-MB-231 at transcript level has been represented in Figure S1B. Moreover, PKM isoform expression at the protein level in SMAR1 overexpressed cells was in concordance with that of transcript level analysis (Fig. 2d). These results suggest that SMAR1 being a tumor suppressor protein, promotes the expression of PKM1 isoform over oncogenic isoform PKM2.

To further validate the role of SMAR1 in *PKM* alternative splicing regulation, a dual reporter *PKM* minigene system has been generated. Schematic representation of dual reporter *PKM* minigene system has been described in Fig. 2e. In this dual reporter *PKM* minigene assay, incorporation of exon 10 leads to eGFP expression whereas incorporation of exon 9 leads to mCherry expression. Fluorescence imaging was done upon ectopic expression of SMAR1 along with the *PKM* minigene system by confocal microscopy to measure eGFP and mCherry expression. Further relative fluorescence intensity was calculated and the fold change of eGFP/mCherry ratio has been calculated. Expression of SMAR1, eGFP, and mCherry was confirmed by western blot (Figure S1C). The fold difference in eGFP/mCherry ratio in SMAR1 overexpression condition was reduced by 2-fold as compared with that of control which further validates its role in *PKM* alternative splicing regulation (Fig. 2f). These results suggest that SMAR1 actively promotes the incorporation of exon 9 and the exclusion of exon 10 leading to higher expression of PKM1 and repression of PKM2 expression in breast cancer cells.

### SMAR1 interacts with PTBP1 and SMAR1-HDAC6 makes a triple complex with PTBP1

**Fig. 3:**
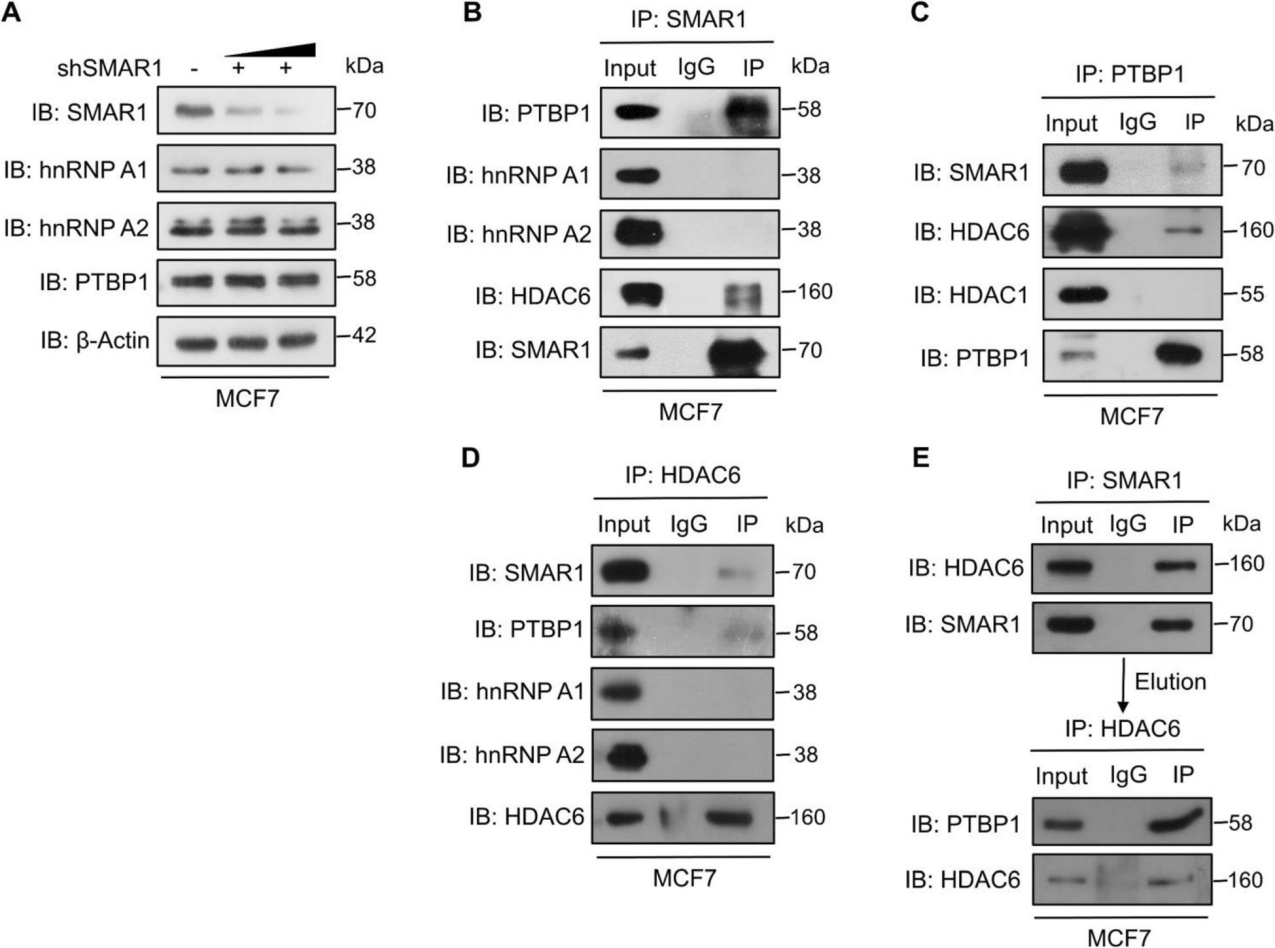
SMAR1 makes a triple complex with PTBP1 and HDAC6. **a** Expression of hnRNP A1, hnRNP A2 and PTBP1 upon shRNA-mediated knockdown of SMAR1 in MCF7. **b** Co-IP of SMAR1 with hnRNP A1, hnRNP A2, PTBP1, and HDAC6 in MCF7 suggest that SMAR1 interacts with PTBP1 and HDAC6. **c** Co-IP of PTBP1 with SMAR1, HDAC1, and HDAC6 in MCF7 suggest that PTBP1 interacts with SMAR1 and HDAC6. **d** Co-IP of HADC6 with hnRNP A1, hnRNP A2, PTBP1, and SMAR1 in MCF7 confirms that HDAC6 interacts with PTBP1 and SMAR1. **e** Sequential Co-IP of SMAR1 with HDAC6 and PTBP1 in MCF7 suggest that SMAR1-HDAC6 makes a triple complex with PTBP1

hnRNP A1, hnRNP A2, and PTBP1 are the three key regulators of *PKM* alternative splicing [11, 12]. When these three proteins are present in higher concentration, they bind to intronic regions flanking exon 9 of *PKM* which leads to the inhibition of incorporation of exon 9 while promoting the incorporation of exon 10, thereby resulting in higher expression of PKM2 and diminished expression of PKM1 [13]. To delineate the detailed molecular mechanism of SMAR1-mediated regulation of *PKM* alternative splicing, expression of hnRNP A1, hnRNP A2, and PTBP1 was measured upon shRNA-mediated knockdown of SMAR1 in MCF7. It was observed that there was no change in the expression of these three splicing regulators upon SMAR1 knockdown (Fig. 3a). This observation eliminates the possibility of transcriptional repression of hnRNP A1, hnRNP A2, and PTBP1 by SMAR1. To determine the role of SMAR1 in the regulation of post-translational modification, the interaction of SMAR1 with these hnRNPs was checked by co-immunoprecipitation (co-IP) and observed that SMAR1 interacts with PTBP1 (Fig. 3b). Interaction of SMAR1 with PTBP1 was further confirmed by reverse co-IP of PTBP1 with SMAR1 (Fig. 3c). SMAR1 is known to regulate various cellular processes with the assistance of HDACs such as HDAC1 and HDAC6 [20, 22, 28, 35]. To identify which HDAC is involved in SMAR1-mediated regulation of *PKM* alternative splicing, the interaction of PTBP1 with HDAC1 and HDAC6 was checked by co-IP which revealed that HDAC6 interacts with PTBP1 (Fig. 3c). Interaction of HDAC6 with PTBP1 and SMAR1 was further confirmed by reverse co-IP of HDAC6 (Fig. 3d). To further investigate the molecular interplay of SMAR1-HDAC6 interaction with PTBP1, sequential IP of SMAR1 and HDAC6 with PTBP1 was performed. Sequential IP experiment revealed that SMAR1-HDAC6 forms a triple complex (Fig. 3e). These results demonstrate that SMAR1 directly interacts with PTBP1 along with HDAC6 and the coexistence of SMAR1-HDAC6-PTBP1 as a ternary complex. This trimeric complex formation further indicates molecular dynamics between these proteins in SMAR1-mediated regulation of *PKM* alternative splicing.

### SMAR1-mediated regulation of *PKM* alternative splicing is HDAC6 dependent

**Fig. 4:**
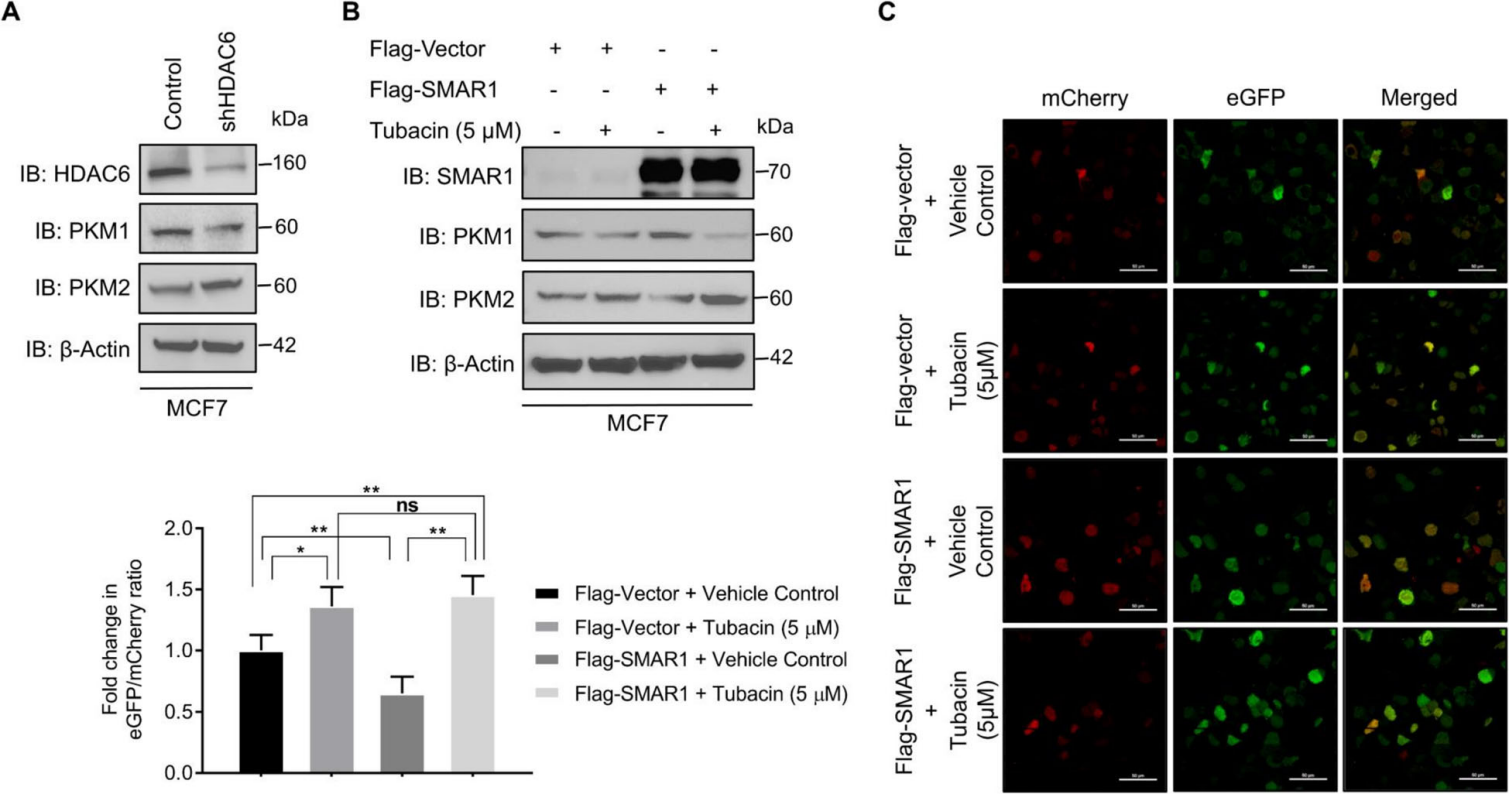
SMAR1-mediated *PKM* alternative splicing is HDAC6 dependent. **a** Expression of PKM isoforms upon shRNA-mediated knockdown of HDAC6 in MCF7 by western blot. **b** Expression of PKM isoforms upon SMAR1 overexpression followed by Tubacin (5 μM) treatment in MCF7 by western blot. **c** Confocal microscopy to check the expression of eGFP and mCherry in MCF7 cells transfected with *PKM* minigene along with Flag-SMAR1. Relative fluorescence intensity of eGFP and mCherry was quantified with the help of ImageJ and fold change in eGFP/mCherry ratio was calculated for control, control + Tubacin (5 μM), Flag-SMAR1 and Flag-SMAR1 + Tubacin (5 μM). Fold change in eGFP/mCherry ratio for SMAR1 overexpression and Tubacin treatment validates the role of HDAC6 in SMAR1-mediated regulation of *PKM* alternative splicing (*n* = 3). Error bars show mean values ± SD. Differences were considered statistically significant with *p < 0.05, **p < 0.01, and ***p < 0.001; *ns* non-significant difference (*p* > 0.05)

To delineate the role of HDAC6 in SMAR1-mediated regulation of *PKM* alternative splicing, expression of PKM isoforms was checked upon shRNA-mediated knockdown of HDAC6 by Western blot. Upon depletion of HDAC6, there was a downregulation in PKM1 expression and an increase in PKM2 expression (Fig. 4a). This indicates the involvement of HDAC6 in *PKM* alternative splicing regulation. To further determine the role of HDAC6 in SMAR1-mediated regulation of *PKM* alternative splicing, SMAR1 overexpressed cells were treated with a specific HDAC6 inhibitor (Tubacin) and PKM isoform expression was checked by western blot. Expression of PKM1 was high and PKM2 was downregulated in the SMAR1 overexpression condition compared with that of control. However, upon Tubacin treatment in SMAR1 overexpressed cells, the expression of PKM1 was reduced and the expression of PKM2 was increased compared with that of only SMAR1 overexpressed cells (Fig. 4b). This confirms that SMAR1-mediated regulation of *PKM* alternative splicing is HDAC6-dependent process. Further validation of the role of HDAC6 in SMAR1-mediated regulation of *PKM* alternative splicing was done by dual reporter *PKM* minigene assay. Fold change in eGFP/mCherry ratio was measured upon SMAR1 overexpression along with Tubacin treatment in cells transfected with *PKM* minigene system compared with the control condition. Fold change in eGFP/mCherry ratio was decreased upon ectopic expression of SMAR1 compared with the control. However, upon tubacin treatment in SMAR1 overexpressed cells resulted in a further increase in the fold change of eGFP/mCherry ratio (Fig. 4c). These observations confirm the role of HDAC6 in SMAR1-mediated regulation of *PKM* alternative splicing. This demonstrates that HDAC6 is actively involved in *PKM* alternative splicing regulation orchestrated by SMAR1.

### SMAR1-HDAC6 deacetylates PTBP1 and modulates its affinity to *PKM* pre-mRNA

**Fig. 5:**
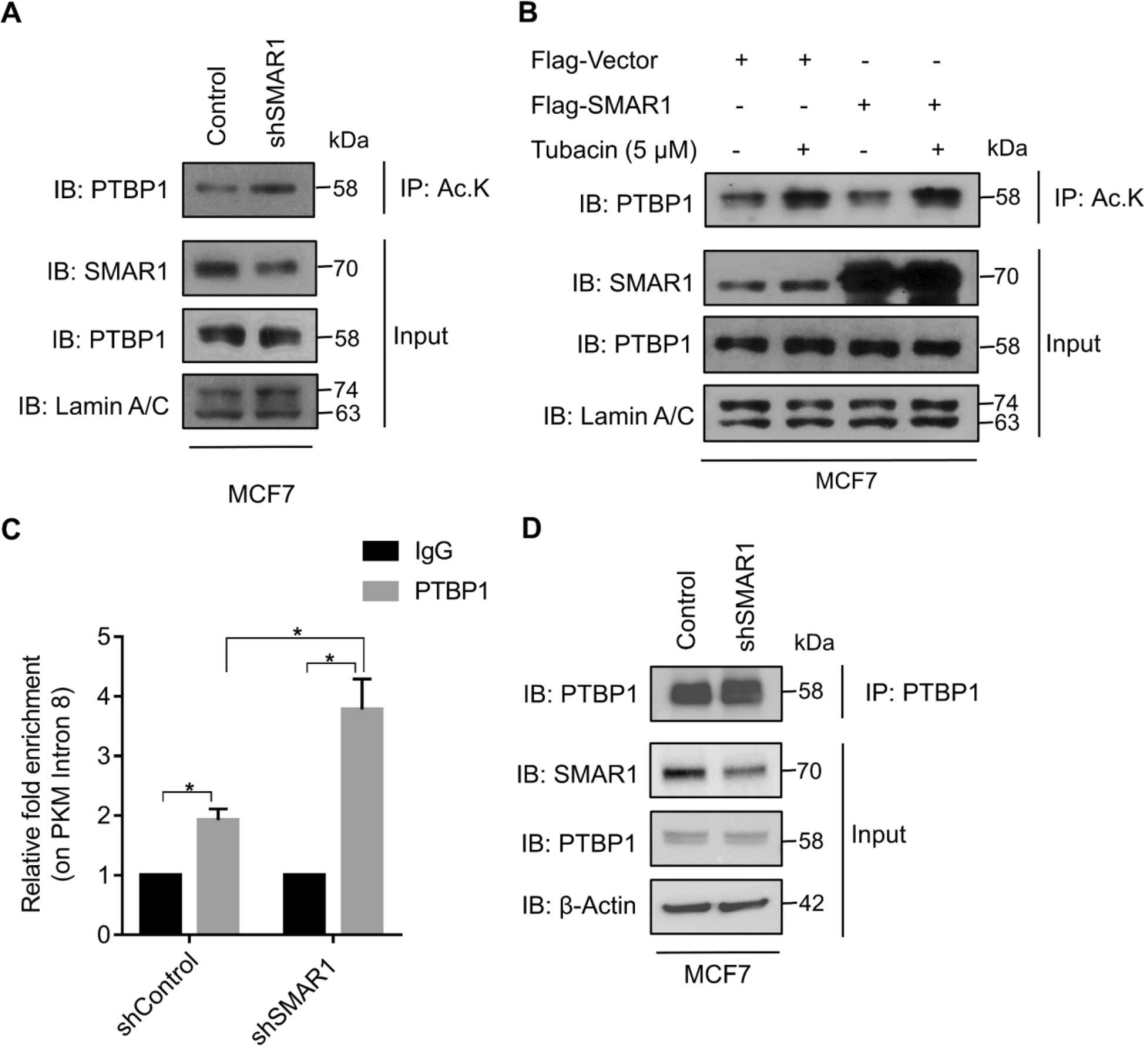
SMAR1-HDAC6 deacetylates PTBP1 and inhibits its binding to *PKM* pre-mRNA. **a** The acetylation status of PTBP1 upon SMAR1 knockdown in MCF7 was checked by IP with an anti-acetyl-lysine antibody. **b** The acetylation status of PTBP1 upon SMAR1 overexpression followed by Tubacin (5 μM) treatment in MCF7 was checked by IP with the anti-acetyl-lysine antibody. **c** CLIP experiment of PTBP1 on intron 8 of *PKM* pre-mRNA in SMAR1 knockdown condition (*n* = 3). **d** IP of PTBP1 to confirm pull down in CLIP experiment upon SMAR1 knockdown in MCF7. Error bars show mean values ± SD. Differences were considered statistically significant with *p < 0.05, **p < 0.01, and ***p < 0.001; *ns* non-significant difference (*p* > 0.05)

Post-translational modifications such as phosphorylation, acetylation, ubiquitination, and sumoylation of splicing factors are known to regulate various alternative splicing events [36–39]. SMAR1 is known to regulate the alternative splicing of *CD44* variants by HDAC6-assisted deacetylation of Sam68 [28]. Moreover, sirtuin-mediated deacetylation of hnRNP A1 has been reported to regulate alternative splicing of the *PKM* gene [40]. To further delineate the role of SMAR1-HDAC6 in the deacetylation of PTBP1, the acetylation status of PTBP1 was checked upon shRNA-mediated depletion of SMAR1 by immunoprecipitation with an anti-acetyl-lysine antibody. In the SMAR1 knockdown condition, there was an increase in the acetylation status of PTBP1 compared with control (Fig. 5a). This observation indicates that the SMAR1 level dictates the acetylation status of PTBP1 and it maintains PTBP1 in a deacetylated state. The acetylation status of PTBP1 was further estimated after SMAR1 overexpression along with Tubacin treatment. Ectopic expression of SMAR1 resulted in a decrease in acetylation status of PTBP1 but upon Tubacin treatment in SMAR1 overexpressed cells, the acetylation status of PTBP1 was restored (Fig. 5b). This suggests that SMAR1 maintains PTBP1 in the deacetylated state in an HDAC6-dependent manner.

To inspect the effect of SMAR1-HDAC6-mediated deacetylation of PTBP1 on its affinity for *PKM* pre-mRNA, UV-crosslinking, and RNA immunoprecipitation (CLIP) of PTBP1 was performed upon shRNA-mediated depletion of SMAR1 in MCF7. Enrichment of PTBP1 on intron 8 of *PKM* pre-mRNA was enhanced by 2-fold in the case of SMAR1 knockdown condition as compared with that of control (Fig. 5c). Immunoprecipitation of PTBP1 in the CLIP experiment was confirmed by western blot (Fig. 5d). Increased enrichment of PTBP1 on *PKM* pre-mRNA in SMAR1 knockdown condition suggests that SMAR1-HDAC6-mediated deacetylation of PTBP1 leads to a reduction in its affinity for *PKM* pre-mRNA and ultimately modulates *PKM* alternative splicing.

### SMAR1 regulates the Warburg effect and breast cancer growth via regulation of PKM2 expression

**Fig. 6:**
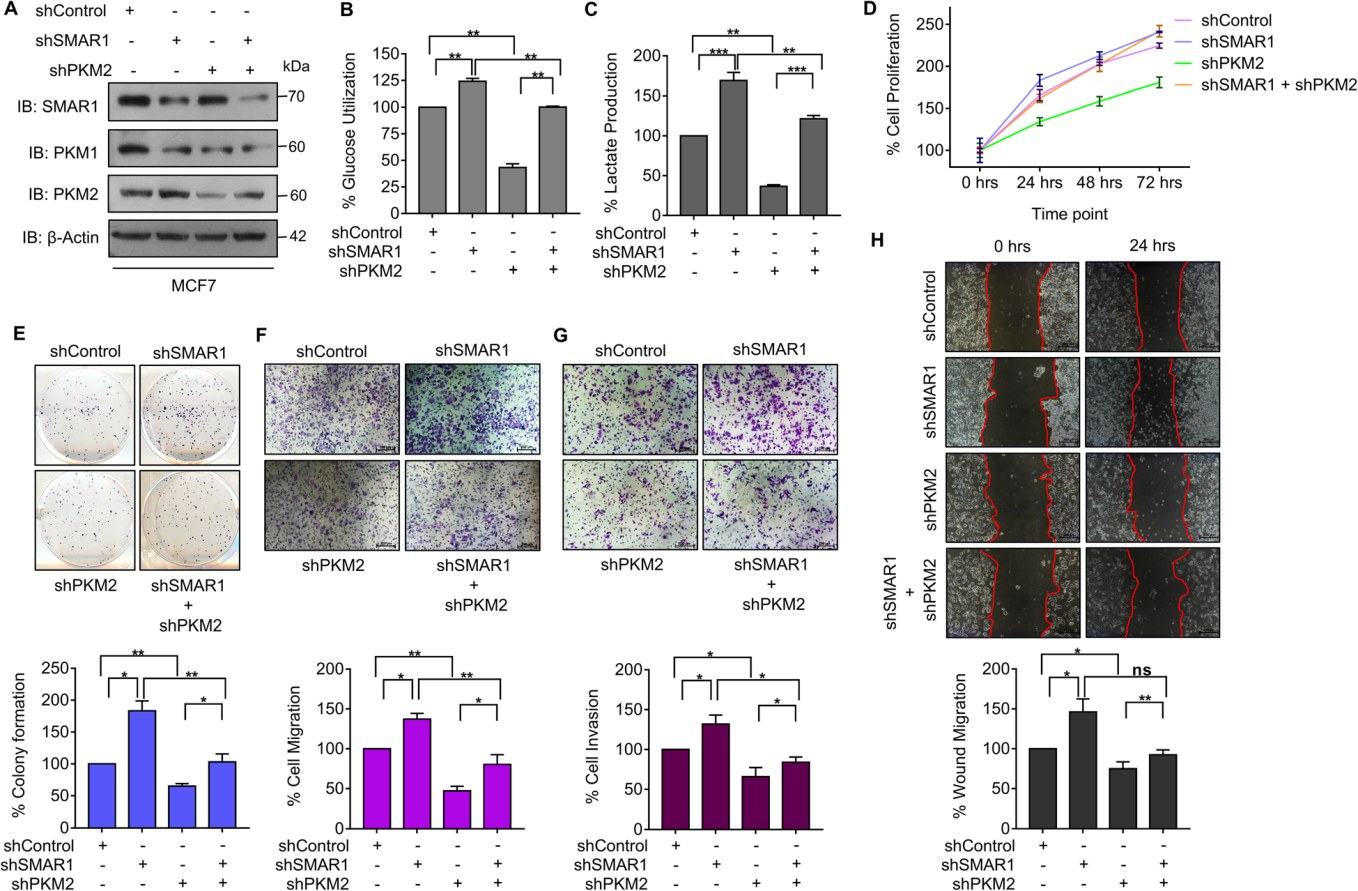
SMAR1 inhibits Warburg effect and tumorigenesis. **a** Expression of PKM isoforms upon shRNA-mediated knockdown of SMAR1 and PKM2 in MCF7. **b** % Glucose utilization was measured in shRNA-mediated knockdown of SMAR1 along with PKM2 in MCF7 by enzymatic assay (*n* = 3). **c** % Lactate formation was measured in shRNA-mediated knockdown of SMAR1 along with PKM2 in MCF7 by enzymatic assay (*n* = 3). **d** % Cell proliferation was measured in shRNA-mediated knockdown of SMAR1 along with PKM2 in MCF7 by MTT assay (*n* = 3). **e** % Colony formation was measured in shRNA-mediated knockdown of SMAR1 along with PKM2 in MCF7 (*n* = 3). **f** % transwell cell migration was measured in shRNA-mediated knockdown of SMAR1 along with PKM2 in MCF7 (*n* = 3). **g** % transwell cell invasion was measured in shRNA-mediated knockdown of SMAR1 along with PKM2 in MCF7 (*n* = 3). **h** % Wound migration was measured in shRNA-mediated knockdown of SMAR1 along with PKM2 in MCF7 (*n* = 3). Error bars show mean values ± SD. Differences were considered statistically significant with *p < 0.05, **p < 0.01 and ***p < 0.001; *ns* non-significant difference (*p* > 0.05)

Higher expression of PKM2 compared with PKM1 is one of the key factors for cancer cells in achieving the metabolic advantage of the Warburg effect compared with normal cells [3]. Reduction in PKM2 expression might lead to inhibition of the Warburg effect and ultimately suppress the tumorigenic potential of cancer cells. SMAR1-mediated regulation of *PKM* alternative splicing leads to suppression of PKM2 and increased expression of PKM1. Based on this observation, we hypothesized that SMAR1 might be playing important role in the regulation of cancer cell metabolism. To determine the role of SMAR1 in the regulation of tumor metabolism, glucose utilization was estimated upon shRNA-mediated decrease of SMAR1 expression along with PKM2 in MCF7. Depletion of PKM2 by shRNA was confirmed by western blot (Figure S2A). Expression of SMAR1 and PKM isoforms upon shRNA-mediated knockdown of SMAR1 along with PKM2 depletion was confirmed by western blot (Fig. 6a). A reduction in SMAR1 expression resulted in increased glucose utilization. Further depletion of PKM2 in SMAR1 knockdown cells resulted in decreased glucose utilization (Fig. 6b). 2-NBDG-mediated glucose uptake upon siRNA-mediated knockdown of SMAR1 validates its role in the regulation of glucose metabolism (Figure S2B). siRNA-mediated knockdown of SMAR1 in MCF7 was confirmed by western blot (Figure S2C). To further decipher the role of SMAR1 in the reversal of the Warburg effect, lactate production was measured in SMAR1 knockdown condition along with PKM2 depletion. SMAR1 depletion condition revealed enhanced lactate production compared with control. Moreover, PKM2 knockdown in SMAR1 depleted cells were having decreased lactate production compared with SMAR1 depleted cells (Fig. 6c). Further, the role of SMAR1 in the regulation of cancer cell metabolism was validated by analyzing the glucose utilization and lactate production upon SMAR1 overexpression in MDA-MB-231 cells. Upon ectopic expression of SMAR1, there was decreased glucose utilization and lactate production compared with that of control (Figure S2D and E). This suggests that SMAR1-mediated regulation of *PKM* alternative splicing inhibits the Warburg effect via suppression of PKM2. These observations reveal the important regulatory role of SMAR1 in keeping cellular metabolism in check via regulation of PKM isoform expression.

PKM2-mediated increase in glucose uptake and lactate production provides a metabolic advantage to cancer cells leading to increased tumor growth [3]. For tumor cells, reprogramming of glucose metabolism and higher glucose utilization are essential for their proliferation and survival [2, 41]. The proliferative potential of cancer cells upon shRNA-mediated depletion of SMAR1 along with PKM2 in MCF7 was assessed by proliferation assay and colony formation assay. In the SMAR1 knockdown condition proliferation rate and colony formation potential were increased which were further reduced due to PKM2 depletion (Fig. 6d and e). These results suggest that SMAR1 inhibits breast cancer growth via reversal of the Warburg effect by suppressing PKM2 expression. Increased lactate production by cancer cells creates acidic surroundings which contribute to the tumorigenic properties of cancer cells such as migration, invasion, and metastasis [42]. Further, the tumorigenic potential of cancer cells upon depletion of SMAR1 along with PKM2 knockdown was measured by in vitro functional assays such as transwell migration, invasion, and wound healing assays. SMAR1 knockdown resulted in an increase in migration, invasion, and wound healing properties of MCF7. Knockdown of PKM2 in SMAR1 depleted cells showed a decrease in migration, invasion, and wound healing ability of cancer cells (Fig. 6f–h). These observations demonstrate that SMAR1 inhibits the tumorigenic potential of cancer cells due to the inhibition of the cancer cell metabolism through the downregulation of PKM2.

### SMAR1 regulates in vivo tumor formation via regulation of PKM2 expression

**Fig. 7:**
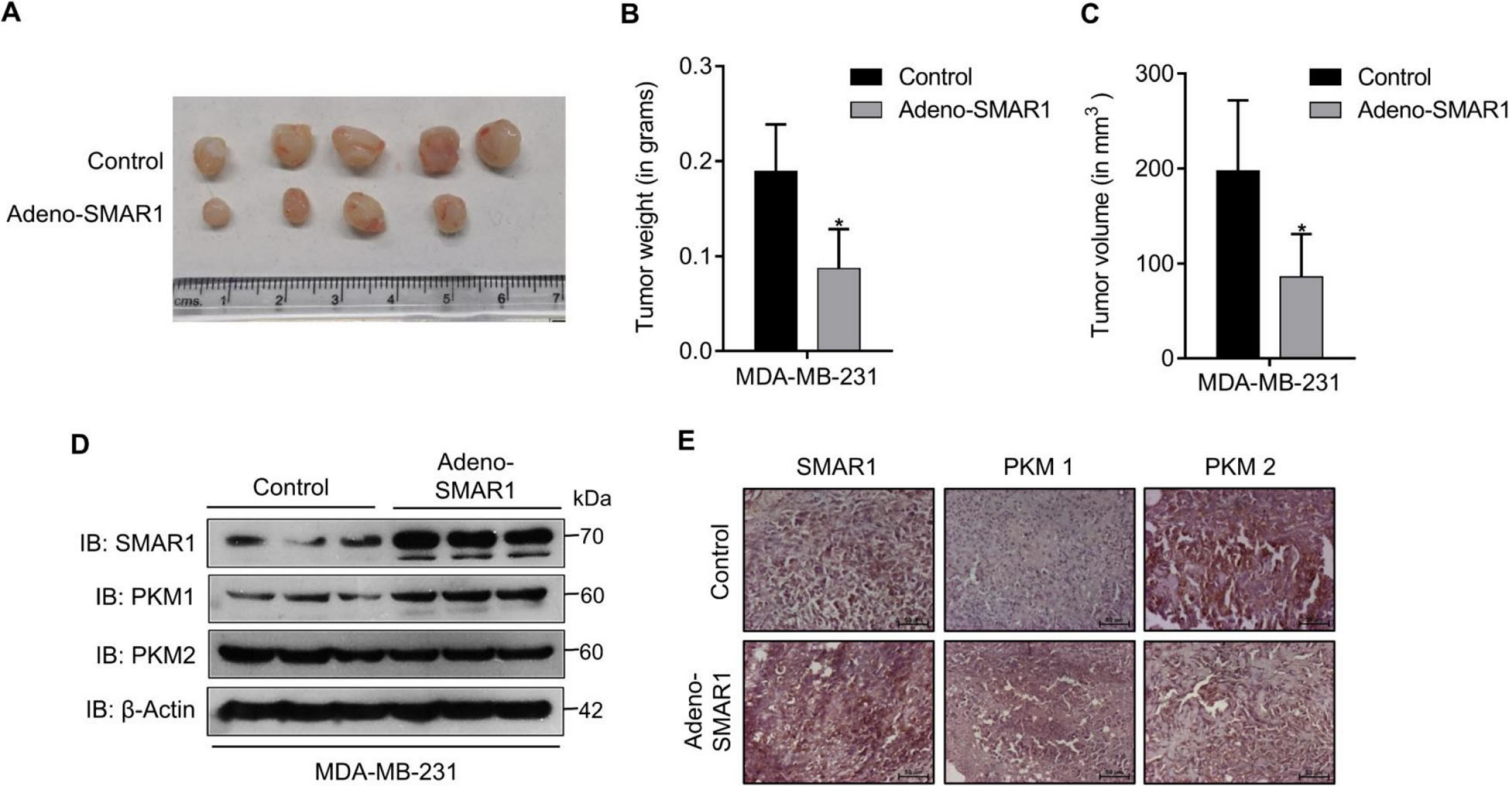
SMAR1 inhibits in vivo tumor formation. **a** Tumors generated in NOD/SCID mice upon injection of adeno-SMAR1-transduced MDA-MB-231 cells compared with control (*n* = 4). **b**, **c** Graphs representing the Tumor weight and Tumor volume. **d**, **e** Expression of PKM isoforms was checked in xenograft samples by western blot and immunohistochemistry. Error bars show mean values ± SD. Differences were considered statistically significant with *p < 0.05, **p < 0.01, and ***p < 0.001; *ns* non-significant difference (*p* > 0.05)

PKM2 contributes to in vivo tumor generation and its progression via regulation of cancer cell metabolism [3]. To identify the role of SMAR1 in tumor formation, tumor xenografts of SMAR1 overexpressed cells were generated by injection of SMAR1-adenovirus-transduced MDA-MB-231 cells in NOD/SCID mice. The tumor burden was compared among SMAR1 overexpressing MDA-MB-231 tumors with that of normal MDA-MB-231 control tumors. In SMAR1 overexpressing group, tumor burden was less compared with that of the control group (Fig. 7a). Moreover, in the SMAR1 overexpression condition, there was a significant reduction in the weight and volume of the tumor compared with control (Fig. 7b and c). These observations indicate that SMAR1 suppresses in vivo tumor generation. A quantitative expression of PKM1 and PKM2 in these xenograft tumor samples was checked by western blot. In adeno-SMAR1 over-expressed tumor samples, the expression of tumorigenic isoform PKM2 was reduced and PKM1 expression was increased compared with control (Fig. 7d). IHC staining of PKM isoforms in these in vivo tumor samples were observed to be correlating with western blot results suggesting higher expression of PKM1 isoform and depleted expression of PKM2 isoform in SMAR1 overexpressing tumors compared with that of control tumors (Fig. 7e). These observations substantiate that SMAR1 suppresses in vivo tumor formation via the regulation of PKM isoform expression.

## Discussion

Here, we describe the detailed mechanism of *PKM* alternative splicing regulation by one of the key tumor suppressor proteins, SMAR1, and its implications in inhibition of cancer cell metabolism and tumorigenesis. SMAR1 has been reported to co-localize with key splicing factor SC35 and it interacts with splicing regulator Sam68 [28]. SMAR1 also contains the RS domain which facilitates its interactions with various snRNAs which are the core components of splicing machinery [28]. Furthermore, SMAR1 regulates alternative splicing of *CD44* variants and *FAS* ligand [28]. This indicates the active involvement of SMAR1 in various alternative splicing events. Our results demonstrate that SMAR1 follows a positive correlation with PKM1 and a negative correlation with oncogenic isoform PKM2 in breast cancer patient samples as well as in breast cancer cell lines. Further experimental approaches have revealed that SMAR1 is actively involved in the regulation of *PKM* alternative splicing. SMAR1 promotes the inclusion of exon 9 and exclusion of exon 10 thus inhibiting the expression of PKM2 which is a tumorigenic isoform of the *PKM* gene. These observations were validated by dual chromatic *PKM* minigene assay, which further confirms the regulatory role of SMAR1 in dictating the outcome of *PKM* alternative splicing. The downregulation of SMAR1 in breast cancer is one of the reasons for enhanced PKM2 expression that leads to altered glucose metabolism and contributes to cancer cell growth.

Nuclear matrix–binding protein, SMAR1 is known to perform various biological functions with help of regulatory proteins such as HDACs [20, 22, 28, 35]. Additionally, various HDACs have been reported to participate in the regulation of alternative splicing through modulation of the acetylation status of histone and non-histone proteins including splicing factors [43]. Earlier studies suggest that SMAR1 inhibits the expression of various genes such as *Cyclin D1*, *BAX*, and *PUMA* by recruiting the repressor complex of HDAC1-mSin3a on the promoter and keeping it in a repressed state by epigenetic modulations [20, 22]. Moreover, SMAR1 also plays a crucial role in deacetylating target proteins such as Ku70 and Sam68 with the help of HDAC6 [28, 35]. This points to the crucial role of SMAR1 in bringing about the post-translational regulation of its target proteins and ultimately dictating their role in various molecular and biological processes. SMAR1-HDAC6-mediated deacetylation of Ku70 dictates cell fate upon exposure to ionizing radiation via regulation of DNA repair and apoptosis [35]. SMAR1 further inhibits metastasis via regulation of *CD44* variants alternative splicing via HDAC6-mediated deacetylation of Sam68 in breast cancer cells [28].

Many post-translational modifications such as phosphorylation, acetylation, ubiquitination, and sumoylation of splicing regulators have been associated with the regulation of various alternative splicing events [28, 36–40]. Previous studies involving *PKM* alternative splicing suggest that three important splicing inhibitors hnRNP A1, hnRNP A2, and PTBP1 regulate *PKM* alternative splicing [11, 12]. Moreover, sirtuin-mediated deacetylation of hnRNP A1 has been reported to regulate alternative splicing of *PKM* gene [40]. This suggests that the acetylation status of splicing factors plays an important role in the regulation of alternative splicing events [28, 40]. Herein, we showed that SMAR1-mediated *PKM* alternative splicing regulation is HDAC6 dependent and SMAR1-HDAC6 forms a triple complex with PTBP1 and maintains it in the deacetylated form. CLIP experiment of PTBP1 in SMAR1 depleted condition resulted in the increased binding of PTBP1 on intron 8 of *PKM* pre-mRNA. This indicates that the binding affinity of PTBP1 is more on *PKM* pre-mRNA in absence of SMAR1. Based on this observation it can be further implied that the deacetylation of PTBP1 by SMAR1-HDAC6 results in the lower affinity of PTBP1 for *PKM* pre-mRNA and thus leading to the inclusion of exon 9 and exclusion of exon 10. This leads to higher expression of PKM1 and lower expression of tumorigenic isoform PKM2. Our findings suggest that SMAR1 brings about HDAC6-mediated deacetylation of PTBP1 and thus maintains PTBP1 in deacetylated form. This deacetylated PTBP1 ultimately leads to the regulation of *PKM* alternative splicing promoting expression of PKM1 and repressing oncogenic isoform PKM2.

Highly proliferating cancer cells gain a metabolic advantage over normal cells via higher expression of PKM2 and exhibits peculiar metabolic properties known as the Warburg effect [2]. Glucose is a primary source of energy and anabolic demands for highly proliferating cancer cells [2]. Moreover, a higher amount of lactate production creates an acidic micro-environment in tumors which favors invasion and metastasis [42]. Our study demonstrates that SMAR1-mediated suppression of PKM2 isoform and upregulation of PKM1 isoform results in inhibition of cancer cell metabolism. SMAR1 inhibits glucose utilization and lactate production via regulation of *PKM* alternative splicing. Our results demonstrate that SMAR1-mediated reversal of the Warburg effect leads to inhibition of cancer cell proliferation, migration, and invasion. Moreover, SMAR1 suppresses in vivo tumor formation via the regulation of PKM isoform expression. This demonstrates the tumor suppressor function of key nuclear matrix–binding protein SMAR1 and its importance in the maintenance of metabolic equilibrium. Future studies on the role of SMAR1 in global alternative splicing regulation might give more insight into the active involvement of tumor suppressor proteins in cancer-associated alternative splicing regulation and its implication in various cellular processes.

In higher grades of cancer, downregulation of SMAR1 expression has been correlated with an increase in tumorigenic potential [21, 23, 24]. SMAR1 expression and functions are also known to be modulated by various cancer-associated signaling pathways including JNK signaling, Wnt signaling, ERK-MAPK pathway [23, 24, 28]. SMAR1 gets highly dysregulated in Wnt signaling–associated colorectal cancer (CRC) [24]. Moreover, activation of ERK-MAPK signaling leads to translocation of SMAR1 to the cytoplasm, resulting in increased *CD44* variants alternative splicing and metastasis in breast cancer cells [28]. A study in the breast cancer model revealed that SMAR1 undergoes proteasomal degradation by Cdc20 in a JNK kinase–dependent manner [23]. Furthermore, microbial peptides that promote SMAR1 stabilization have been correlated with inhibition of Wnt/β-catenin activities in CRC [24]. A study involving stabilization of SMAR1 expression by treatment with isothiocyanate derivative has been co-related with anti-HIV activity [44]. Our findings reveal that SMAR1 knockdown promotes the Warburg effect and tumorigenic potential of breast cancer cells by modulating PKM isoform expression indicating an important regulatory role of tumor suppressor protein in cellular homeostasis. This indicates that future studies targeting cancer cell metabolism and cancer progression via stabilization of SMAR1 expression might lead to better therapeutic efficacy in cancer treatment.

## Conclusion

Our study in the breast cancer model highlights that nuclear matrix–binding protein SMAR1 regulates *PKM* alternative splicing and inhibits the expression of PKM2 and promotes the expression of PKM1. SMAR1 regulates *PKM* alternative splicing via deacetylation of a key alternative splicing inhibitor PTBP1 in an HDAC6-dependent manner. Further, in vitro enzymatic assays, functional assays, and in vivo tumor model suggest that SMAR1 inhibits tumor cell metabolism and tumorigenic properties of cancer cells by suppressing the expression of tumor-associated isoform PKM2 and promoting the expression of enzymatically more efficient PKM1 isoform.

## Supplementary Information

**Additional file 1: Figure S1.** (A) shRNA-mediated knockdown of SMAR1 in MCF7 by qRT-PCR. (B) Flag-SMAR1 mediated overexpression of SMAR1 in MDA-MB-231 by qRT-PCR. (C) Expression of eGFP/mCherry upon SMAR1 overexpression in MCF7. Error bars show mean values ± SD. Differences were considered statistically significant with *p < 0.05, **p < 0.01 and ***p < 0.001, ns non-significant difference (p > 0.05).

**Additional file 2: Figure S2.** (A) shRNA-mediated PKM2 knockdown in MCF7. (B) % Glucose uptake (2-NBDG) upon siRNA-mediated knockdown of SMAR1 in MCF7 by FACS. (C) Expression of PKM isoforms upon siRNA-mediated knockdown of SMAR1 in MCF7 by western blot. (D) % Glucose utilization was measured upon Flag-SMAR1 mediated overexpression of SMAR1 in MDA-MB-231 by enzymatic assay (n=3). (E) % Lactate formation was measured upon Flag-SMAR1 mediated overexpression of SMAR1 in MDA-MB-231 by enzymatic assay (n=3). Error bars show mean values ± SD. Differences were considered statistically significant with *p < 0.05, **p < 0.01 and ***p < 0.001, ns non-significant difference (p > 0.05).

**Additional file 3: Table S1.** List of primer sequences utilized for qRT-PCR.

## Data Availability

All data generated or analyzed during this study are included in this published article and its supplementary information files.

## Abbreviations

ATP: Adenosine triphosphate
CLIP: UV-crosslinking and RNA immunoprecipitation
CRC: Colorectal cancer
HDAC6: Histone deacetylase 6
hnRNP: Heterogeneous nuclear ribonucleoprotein
IP: Immunoprecipitation
MAR: Matrix-associated region
MARBP: Matrix-associated region-binding protein
PKM: Pyruvate kinase muscle
PTBP1: Polypyrimidine tract–binding protein 1
SMAR1: Scaffold matrix–associated region-binding protein 1
TCA: Tricarboxylic acid
