# Supplementary figures and images for: Tumor suppressor SMAR1 regulates PKM alternative splicing by HDAC6-mediated deacetylation of PTBP1
Source: Cancer Metab. 2021 Apr 16;9:16. doi: 10.1186/s40170-021-00252-x (PMC8052847; doi:10.1186/s40170-021-00252-x)

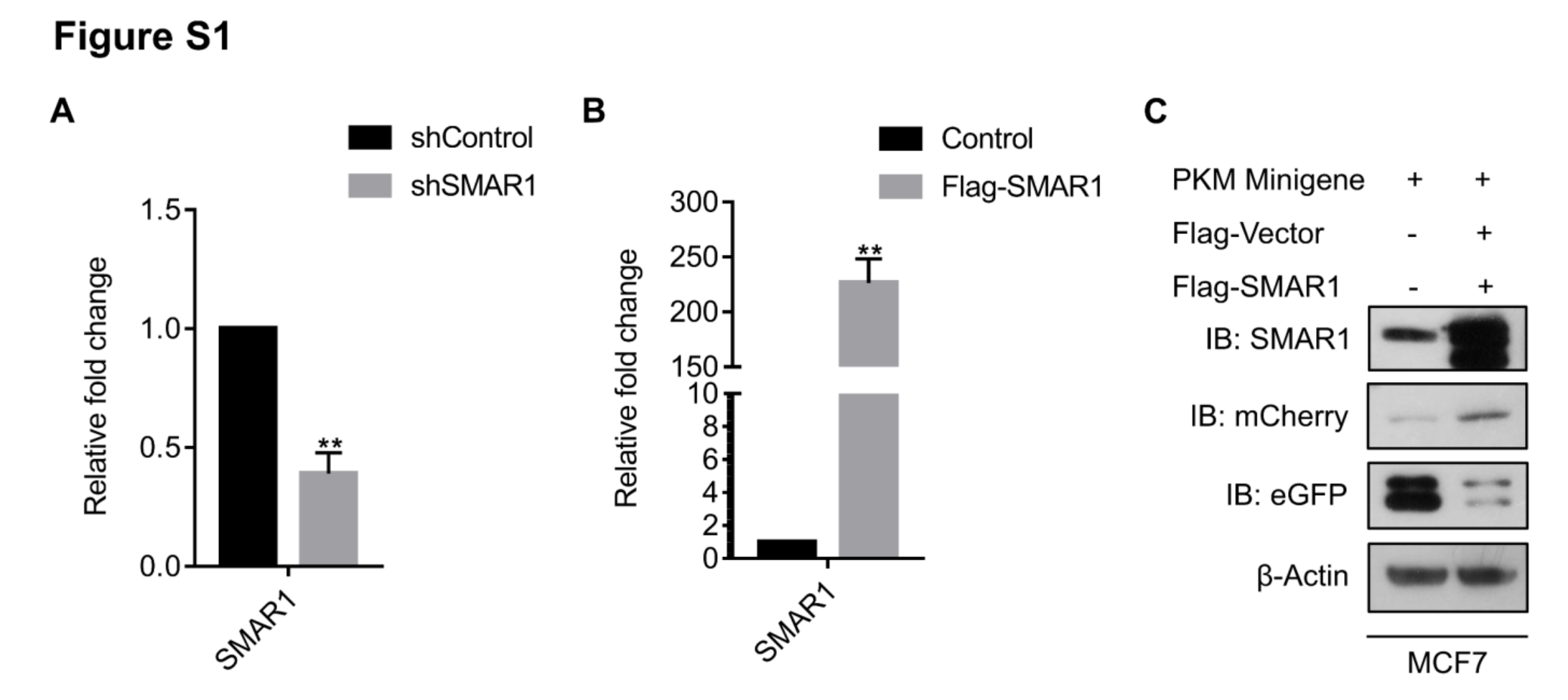

Supplement: Supplementary file 1 — Additional file 1: Figure S1. (A) shRNA-mediated knockdown of SMAR1 in MCF7 by qRT-PCR. (B) Flag-SMAR1 mediated overexpression of SMAR1 in MDA-MB-231 by qRT-PCR. (C) Expression of eGFP/mCherry upon SMAR1 overexpression in MCF7. Error bars show mean values ± SD. Differences were considered statistically significant with *p < 0.05, **p < 0.01 and ***p < 0.001, ns non-significant difference (p > 0.05). [file 40170_2021_252_MOESM1_ESM.tif]

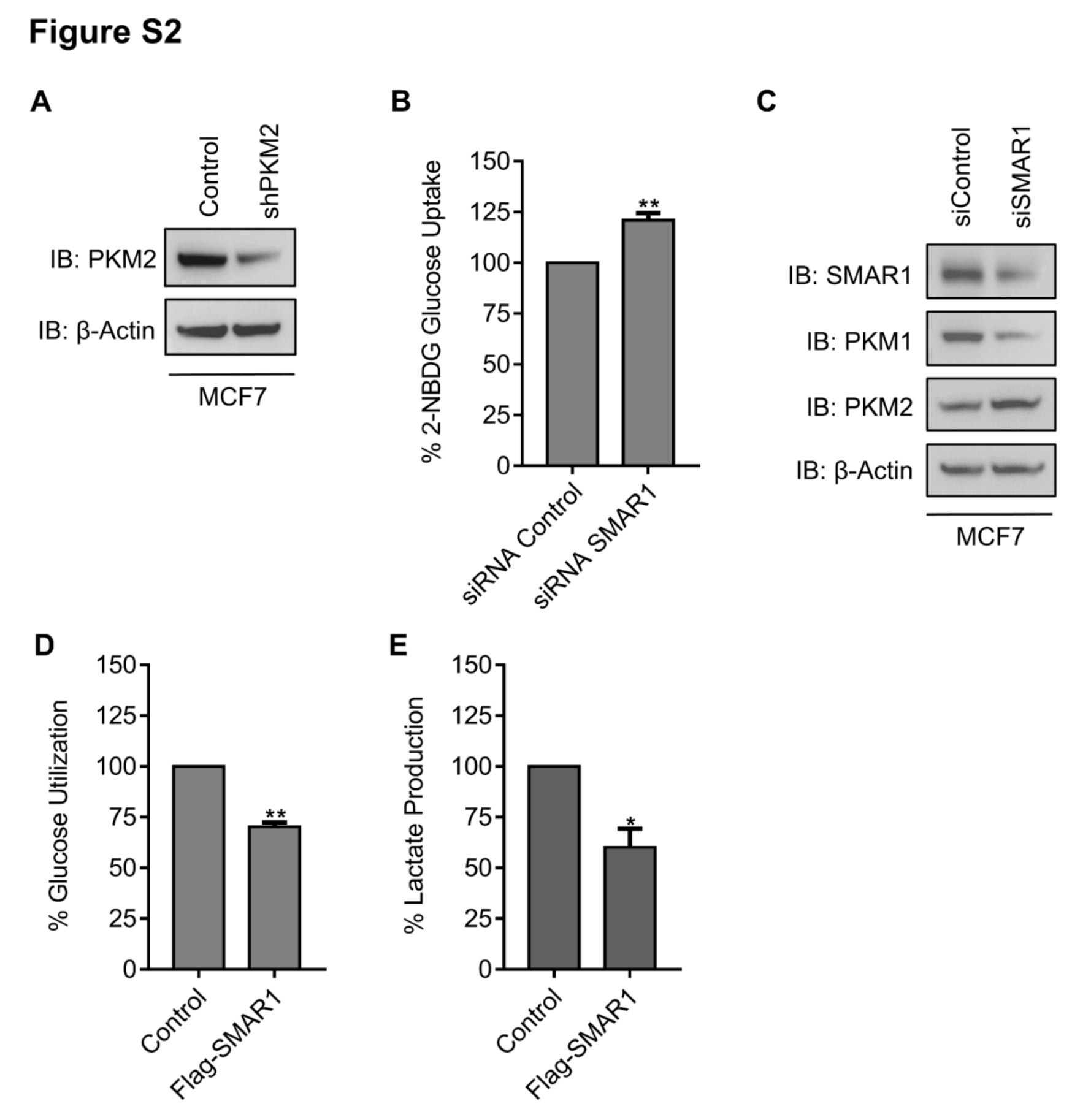

Supplement: Supplementary file 2 — Additional file 2: Figure S2. (A) shRNA-mediated PKM2 knockdown in MCF7. (B) % Glucose uptake (2-NBDG) upon siRNA-mediated knockdown of SMAR1 in MCF7 by FACS. (C) Expression of PKM isoforms upon siRNA-mediated knockdown of SMAR1 in MCF7 by western blot. (D) % Glucose utilization was measured upon Flag-SMAR1 mediated overexpression of SMAR1 in MDA-MB-231 by enzymatic assay (n=3). (E) % Lactate formation was measured upon Flag-SMAR1 mediated overexpression of SMAR1 in MDA-MB-231 by enzymatic assay (n=3). Error bars show mean values ± SD. Differences were considered statistically significant with *p < 0.05, **p < 0.01 and ***p < 0.001, ns non-significant difference (p > 0.05). [file 40170_2021_252_MOESM2_ESM.tif]
