# Supplementary material for: Tumor suppressor SMAR1 regulates PKM alternative splicing by HDAC6-mediated deacetylation of PTBP1
Source: Cancer Metab. 2021 Apr 16;9:16. doi: 10.1186/s40170-021-00252-x (PMC8052847; doi:10.1186/s40170-021-00252-x)
Supplement: Supplementary file 3 — Additional file 3: Table S1. List of primer sequences utilized for qRT-PCR. [file 40170_2021_252_MOESM3_ESM.pdf]

Additional File 3: Table S1: List of primer sequences utilized for qRT-PCR.

Sr. No.	Primer	Sequence (5’-3’)
1	SMAR1 Fwd	CTTGCGGTTGGATAGCATTGA
2	SMAR1 Rev	GCTGCTTGTTCGTGACCAGAT
3	PKM1 (PKM E8-E9) Fwd	ATGCAGCACCTGATAGCTCGTGA
4	PKM1 (PKM E9) Rev	TGCCAGACTCCGTCAGAACTATCA
5	PKM2 (PKM E10-11) Fwd	TCACCAAGTCTGGCAGGTCTG
6	PKM2 (PKM E11) Rev	CATTCATGGCAAAGTTCACCCGGA
7	PKM E11 Fwd	CCATCATTGCTGTGACCCGGAAT
8	PKM E11 Rev	CATTCATGGCAAAGTTCACCCGGA
9	18S rRNA Fwd	CTACCACATCCAAGGAAGCA
10	18S rRNA Rev	TTTTTCGTCACTACCTCCCCG
11	PKM Intron 8 (PTBP1 binding site) Fwd	TGTTGTGTCTCGTTTTTTTCCTCCTCC
12	PKM Intron 8 (PTBP1 binding site) Rev	CTCACGAGCTATCTGTAAGGTTTAGG
